# Exploring behavioral factors and emotional mechanisms underlying older adult users' adoption of smart health services: evidence from PLS-SEM and fsQCA

**DOI:** 10.3389/fpubh.2025.1673340

**Published:** 2025-09-26

**Authors:** Ming-Xi Sun, Zhi-Feng Zhao

**Affiliations:** School of Arts, Soochow University, Suzhou, China

**Keywords:** emotionally durable design, TAM, smart health services, PLS-SEM, fsQCA

## Abstract

**Introduction:**

In a population aging society, home-based older adult care as the primary model have experienced a robust growth in demand for smart health services (SHS), reflecting an active response to evolving older adult care needs. However, older adult users' adoption rates are constrained by the interplay of technological and emotional factors, with the underlying mechanisms remain unclear.

**Methods:**

To address this concern, this study integrates the Technology Acceptance Model (TAM) and Emotional Durable Design (EDD) theory to uncover the behavioral factors and emotional driving mechanisms behind older adult users' adoption of SHS. A total of 244 valid questionnaires from older adult users were collected. Partial Least Squares Structural Equation Modeling (PLS-SEM) was employed to analyze the net effects of variables, combined with fuzzy-set Qualitative Comparative Analysis (fsQCA) to identify configurational paths.

**Results and discussion:**

The results show that perceived ease of use, perceived usefulness, and perceived emotion directly and positively influence behavioral intention. Mental interaction, self-actualization, and dynamic adaptation in emotional durable design exert indirect effects on behavioral intention through internalization processes. fsQCA identified 7 configurational paths leading to high behavioral intention, confirming the combined effects of “technology-emotion” factors, with no single factor constituting a necessary condition. This study contributes to expanding the emotional explanatory dimension of TAM, providing a theoretical foundation and practical guidelines for the human-centered design and promotion of SHS.

## 1 Introduction

In recent decades, growing families are confronted with the challenge of caring for older adult relatives, particularly those living alone. According to the Seventh National Population Census, for instance, the population aged 60 and above in China has reached 264 million, accounting for 18.7% of the total population. Both the size of the older adult population and its proportion within the total population continue to rise, indicating that China is on the verge of entering a moderately aging society (1). Concurrently, driven by the miniaturization of family structures and the expansion of the empty-nest older adult group, home-based older adult care services supported by community resources have gradually become a mainstream model in the global older adult care system (2). In China, a care model centered on families and communities has taken shape, with 90% of the older adult opting for home-based care services and 7% choosing community care (3). As population aging intensifies, the demand for home-based older adult care services is growing rapidly (4). In this context, how to leverage technological innovation to empower home-based older adult care scenarios and assist the older adult in achieving health management and life convenience has become an urgent social issue.

Technological advancements in the Internet of Things, big data, and artificial intelligence are driving significant transformations in older adult care, particularly through the shift from traditional healthcare models to digitalized smart health services (SHS) in older adult care communities (5–7). SHS involves the integration of next-generation intelligent technologies into traditional healthcare industries, aiming to provide patients with more convenient, flexible, personalized, and diversified medical services (8). By rigorously and continuously monitoring physiological and activity characteristics in users' daily lives, SHS can offer precision health management support for the older adult. Despite the evident benefits of smart health service management for the older adult, over 60% of users aged 50 and above perceive that existing product designs fail to account for their age-related needs (9). As a mature, technology-evolving system (10), SHS has been studied in terms of product/service attributes and user characteristics: Ji et al. (11) explored older adult chronic disease patients' acceptance of care technologies; Sintonen and Immonen. (12) investigated willingness toward remote care; and Körtner et al. (13) analyzed interaction factors with home service products, with additional research on user differences like age stratification (14). Practically, SHS connects hospitals, doctors, patients, and families, providing diverse services such as remote prescriptions, telemedicine, disease detection and prevention, as well as facilitating the use of related smart health products and support, to meet the healthcare needs of different populations (15). supports P4 (Predictive, Participatory, Personalized, and Preventive) medicine for lifelong healthcare (13), and has been applied in cases such as South Korea's SHHS during the COVID-19 pandemic (16) and AI-driven applications (e.g., Ada App) offering tailored services (15).

However, technological advancements such as telemedicine, digital healthcare, and artificial intelligence (AI) hold little value if the older adult cannot leverage them to benefit their health. despite the multiple advantages of smart health services, their successful implementation hinges largely on the target group's acceptance of both the technology and its delivery model (15). Existing research on smart health services predominantly explored service models (17), technical feasibility (18), and service frameworks (19); it has overemphasized acceptance of technological functions while underappreciating the central role of emotional value in users' decision-making processes (10, 20). From the perspective of user experience, emotional value in products and services is often more appealing to users than the technology itself (21). Furthermore, users' willingness to adopt SHS is a process characterized by complex causal relationships, highlighting the importance of complementary research on the configurational effects between factors from a holistic perspective (22). However, previous studies have primarily focused on the “net effects” between variables, understating the complexity of variable causal configurations. The triggering factors of SHS on users' willingness to use remain unclear. Therefore, we propose the following research questions:

RQ1: What antecedents influence older adult users' adoption of SHS from the perspective of emotional durability?

RQ2: What are the underlying mechanisms among these antecedent factors in shaping older adult users' SHS adoption behavior?

To address the research questions, this study integrates Emotional Durable Design theory with the TAM framework, employing Partial Least Squares Structural Equation Modeling (PLS-SEM) and fuzzy-set Qualitative Comparative Analysis (fsQCA) to explore the key factors influencing older adult users' acceptance of SHS and their configurational pathways. This research attempts to make two theoretical contributions. Firstly, by deconstructing the interaction mechanisms between technological attributes and emotional elements, it aims to elucidate a multidimensional driving model of older adult users' behavioral decisions, explore how emotional factors influence older adult users' focus of attention in their willingness to use from the perspective of user experience, and further propose a dual-dimensional (technology-emotion) oriented design framework. Specifically, we examine the technological and emotional dimensions of SHS, with a focus on how emotional durability factors influence users' willingness to use. Secondly, by combining PLS-SEM and fsQCA methods, this study not only examines the net effects of individual variables but also reveals, from the perspective of causal configuration, the complex combinations and interactions of different variables and their comprehensive impact on users' willingness to use. This overcomes the oversimplification issues in hypothesis testing of traditional regression analyses. It is expected that findings may deepen emotional value's explanatory power in technology acceptance theory and provide practical references for humanized SHS, potentially improving the older adults health management and life wellbeing.

Our findings reveal that the “smart” core of SHS should undergo a paradigm shift from technology-centric to user-centric. This means that the development of products and services must be rooted in the actual needs and acceptance thresholds of the older adult, breaking through the “instrumental” limitations of traditional smart older adult care and constructing more life-immersive services through emotional durable design. Such a design transformation, which infuses emotional warmth into technological carriers, can effectively shorten the emotional distance between users and technology (3), and is of significant value in enhancing older adult users' technological identification.

The remainder of this paper is structured as follows: Section 2 introduces relevant theories and studies through a literature review, proposes a conceptual model to explain older adult users' behavioral intentions regarding SHS, and puts forward 11 hypotheses. Section 3 reports the research methodology and data collection, while Section 4 conducts data analysis and model validation. In Section 5, we discuss the research findings to deepen the understanding of SHS behavioral intentions, elaborate on the limitations of this study, and provide suggestions for future work. Finally, the paper concludes with a summary.

## 2 Hypothesis development

Technology Acceptance Model (TAM)—a classic framework for explaining and predicting individuals' technology acceptance behavior, is widely used to assess the likelihood of consumers accepting or rejecting new technologies (23, 24). The theory is proposed by Davis, which draws inspirations from the Theory of Reasoned Action (TRA) (25) and is also related to Ajzen's Theory of Planned Behavior (TPB) (26). TAM embraces structural simplicity, which includes only two core influencing factors: perceived usefulness (PU) and perceived ease of use (PEOU). However, this simplicity becomes a limitation, as it underappreciates other determinants of technology acceptance (27). Notably, TAM allows researchers to integrate context-specific constructs to develop modified models, which better elucidate technology acceptance in specific domains (28). This adaptability, inclusiveness, and extensibility have made it widely applicable for examining users' acceptance of software and technologies, aiding in predicting new technology adoption willingness (29). The model has been tested multiple times in the healthcare field, especially widely applied in studies on older adult individuals' technology acceptance, and can effectively explain why people choose to use new technologies (30).

To comprehensively explain new technology acceptance, researchers have integrate additional influencing factors into TAM to enhance its contextual relevance and prediction accuracy (31). Examples include TAM2 (32), the Unified Theory of Acceptance and Use of Technology (UTAUT) (33, 34), the extended UTAUT2 (35), and hybrid models that combine TAM with other predictors. Hybrid models not only consider perceived usefulness and perceived ease of use but also introduce other factors such as enjoyment (36), perceived surprise (37), and information trust (38). Furthermore, the factors influencing intention continue to expand, further enriching our understanding of the mechanisms underlying technology acceptance. These models provide researchers with tools to more accurately capture the opinions and attitudes of potential users. Specifically, in healthcare technology and healthcare IT adoption, TAM is widely accepted as a relevant model for identifying factors affecting the adoption of IT in healthcare, as well as users' perceptions and behaviors (39, 40).

Emotionally Durable Design (EDD), first proposed by Chapman in foundational research on sustainable products (41), centers on establishing emotional attachment between users and products through design to extend product lifecycles. This theory transcends the traditional design paradigm centered on physical performance and shifts to focusing on emotional connections between subjects and objects in six relational spaces (cognition, experience, memory, etc.) (42). Haines-Gadd further expanded the EDD theoretical system (43), constructing an analytical framework containing nine dimensions, emphasizing that emotional narratives drive the product development process. However, existing studies mostly focus on single-dimensional analysis, which are often limited by the factors considered in these studies and lack the integration of multi-dimensional relational spaces (44). In addition, affective computing focuses on identifying and responding to user emotions to optimize human-computer interaction, and our research further explores how the three dimensions of EDD positively shape emotional perception rather than simply responding to these emotions. This complements the research by Shimizu (20), who found that affective value is a key unmet need in older users when receiving SHS.

**Figure 1:**
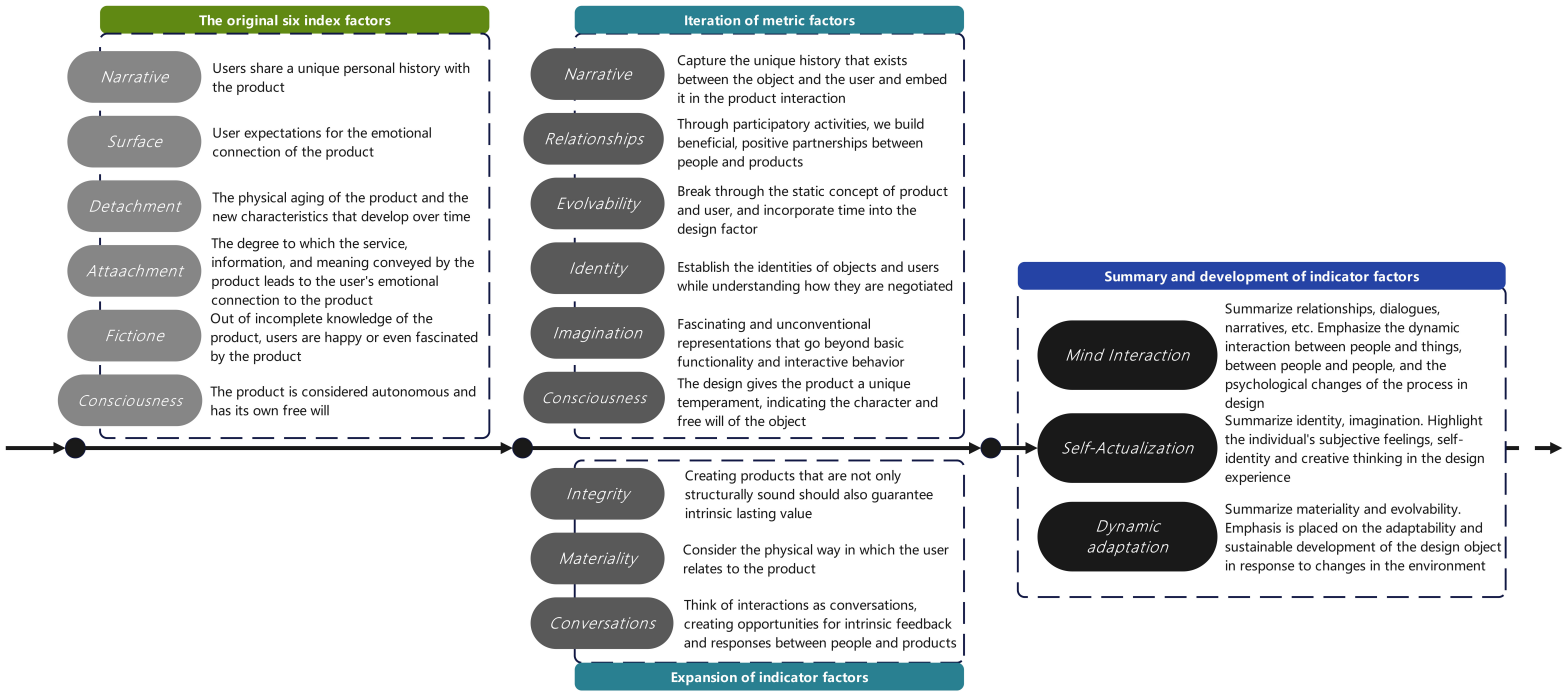
Three-dimensional EDD mapping model.

Building on existing theories, this study proposes a three-dimensional EDD mapping model (Figure 1): Mind Interaction (MI), Self-Actualization (SA), and Dynamic Adaptation (DA). MI covers relationship construction, narrative transmission, and two-way dialogue mechanisms, focusing on describing dynamic interactions and psychological changes between people, between people and objects, and those reflected in the design process; SA includes three levels: identity, value perception, and creative participation, highlighting individuals' subjective feelings, self-identity, and creative thinking in the design experience; DA emphasizes the environmental adaptability of products, involving consciousness guidance and evolutionary ability to achieve sustainable development, with a focus on the adaptability and sustainable development ability of design objects in response to environmental changes. MI, SA, and DA have been discussed in related fields (45–49). It should be emphasized that this study aims to realize the integration of the three, with special attention to the dynamic coupling relationships between various dimensions and their synergistic effects in smart health service scenarios. Specifically, MI focuses on the interaction methods in the development of the relationship between users and SHS, SA focuses on users' thinking triggered by the interaction process, and DA focuses on the adaptability of design objects to changes based on feedback.

Technology acceptance is defined as the psychological determinant of users' behavioral intention to use a specific technology, regardless of prior experience or actual usage. Building on previous work, we describe the willingness to use Smart Health Services (SHS) as an emotionally durable realization process embedded in these behaviors. Existing measurement items in the field of SHS have not comprehensively incorporated emotional durability factors. The model in this study consists of the original TAM model and four additional constructs, including perceived usefulness (PU), perceived ease of use (PEOU), behavioral intention (BI), perceived emotion (PE), mental interaction (MI), self-actualization (SA), and dynamic adaptation (DA). Among them, BI is the dependent variable, and the others are independent variables.

**Figure 2:**
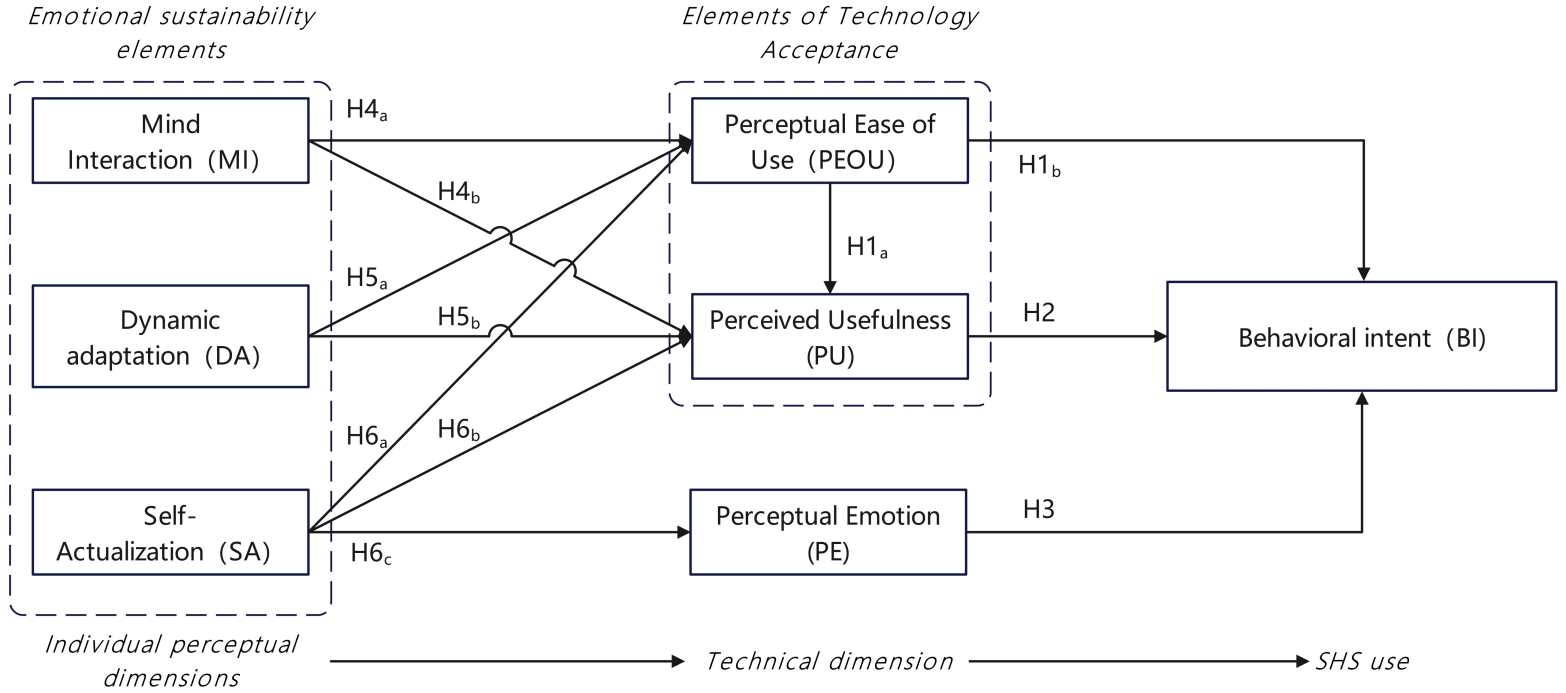
Hypothesis model.

Theoretically, emotional durability theory complements the understanding of how users' acceptance is shaped by perceived technological realization. Specifically, mental interaction (MI), self-actualization (SA), and dynamic adaptation (DA) function as external stimuli; PU, PEOU, and PE represent the internalization process of these stimuli (serving as the “organism” in the model); and willingness to use reflects users' response to technology acceptability, i.e., the outcome formed after external stimuli are internalized. In summary, this study proposes a model based on TAM and EDD to explore users' willingness to use (Figure 2), aiming to understand how older adult users' willingness to use SHS is formed through technological and emotional dimensions. In the following, we describe the hypothesis development in detail.

### 2.1 Behavioral intent

One of the key components of TAM is behavioral intention (BI), which refers to the intention to use or adopt a technology, influenced by an individual's attitude toward using that technology. In the original TAM, BI is defined as “the degree to which an individual formulates a conscious plan to perform or not perform certain specific behaviors in the future,” and it has been demonstrated that an individual's behavioral intention to use a technology exerts a positive impact on their actual use of the technology (23, 50). Consequently, numerous studies have focused on behavioral intention to use technology as a means to predict actual usage and technology acceptance (10, 34, 51). Additionally, perceived usefulness is specified to have an independent effect on behavioral intention (BI). Some studies have reported that the relationship between perceived usefulness (PU) and the intention to use or actual use of health services is statistically significant (51, 52), while in other studies, the PEOU-BI relationship was found to be statistically non-significant (53). According to TAM, an individual's behavioral intention to use a technology, attitude toward use, and actual use are influenced by two primary predictors: perceived usefulness and perceived ease of use. Previous research has confirmed that users' intention to use a technology positively affects their actual use of it. Therefore, in this study, behavioral intention is identified as the dependent variable for predicting the actual usage of the technology.

### 2.2 Perceived ease of use and perceived usefulness

In this study, perceived ease of use (PEOU) is defined as “the extent to which older adult users feel familiar and uncomplicated when using products and services,” while perceived usefulness (PU) refers to “older adult users' subjective evaluation of whether products and services possess real value and the degree to which they assist in achieving their personal goals compared to other products.” The TAM model has been validated in numerous studies, capable of explaining individuals' intention to accept technologies in intelligent care systems (34, 51, 52). Based on this, it is suggested that users will accept a new technology when their perception of its usefulness or advantages is significantly positive. TAM assumes that an individual's perception of the ease of access to a technology influences their evaluation of its usefulness (50). The basic structure of the model includes perceived ease of use (PEOU), perceived usefulness (PU), and behavioral intention to use (BI). Within this model, both PU and PEOU exert a positive impact on BI. Additionally, PEOU can enhance PU. Based on the above, we propose the following hypotheses:

H1: Perceived ease of use is positively correlated with (a) perceived usefulness and (b) behavioral intention.
H2: Perceived usefulness is positively correlated with behavioral intention.

### 2.3 Perceptual emotion

Perceived emotion (PE) is defined as “the possibility of perceiving the emotion of a whole through perceiving one or more parts of that whole” (54). Older adult individuals often experience a sense of alienation and uncertainty toward digital tools during the technology acceptance process, while positive perceived emotions (such as pleasure, a sense of being cared for, and a sense of security during use) can effectively alleviate such psychological resistance and lower the psychological threshold for technology usage (55). When older adult users can perceive the humanistic care embedded in the service, this emotional experience will strengthen their recognition of the service's value through the emotional transfer effect, making them more inclined to regard the service as a reliable partner in meeting their health needs (11) rather than a cold technical tool, thereby enhancing their willingness to use it. Furthermore, when they perceive the respect and care conveyed by the service's meticulous considerations in aspects such as data protection and service response, their concerns about risks will be significantly reduced (56), thus strengthening their confidence in continuous use. Based on this, we propose the following hypothesis:

H3: There is a positive correlation between perceived emotion and behavioral intention.

### 2.4 Mind interaction

Mind Interaction (MI) is defined as a dynamic psychological connection process formed through relationship construction, narrative transmission, and two-way dialogue among interacting subjects (including individuals, groups, and technical systems) (47). Its core lies in transcending superficial behavioral interactions to achieve the dynamic integration of information exchange and value identification. This interaction mechanism breaks through the one-way information transmission mode of traditional technical interactions, emphasizing the collaborative evolution of both interacting parties at the cognitive, emotional, and behavioral levels (45). Through continuous meaning co-construction and feedback regulation, it achieves a deep psychological alignment.

The perception of the usefulness of smart health services depends on users' judgment of “whether the service can solve their own problems,” as users can clearly recognize the practical value of the service in improving health autonomy and reducing family care pressure (57). However, older adult individuals often develop psychological resistance during technology use due to excessive cognitive load or unfamiliar interaction logic. When users can communicate their cognitive habits and expression preferences through two-way dialogue, they can perceive the controllability and understandability of the interaction process, thereby reducing the sense of alienation from the technology. For example, the service dynamically adjusts interaction methods based on user behavior feedback (58), and explains health data using narrative language that matches user behavior (59, 60). Additionally, previous studies have shown that the easier users find it to interact and communicate with products and services, the more they perceive these products and services as highly helpful to their living environment (15, 51). Based on this, we propose the following hypotheses:

H4: There is a positive correlation between mental interaction and (a) perceived ease of use and (b) perceived usefulness.

### 2.5 Dynamic adaptation

Dynamic adaptation (DA) is defined as a process in which individuals or systems continuously adjust their own states, behavioral patterns, and cognitive styles in response to changes in internal and external environments, so as to better meet environmental needs and achieve expected goals (48, 61). When smart health services can capture older adult users' digital skill levels, operational habits, and physiological characteristics (such as eyesight and hand flexibility), respond to the heterogeneous needs of older adult users (49), and personally adjust service interfaces (such as font size and simplified operation steps), interaction methods (such as voice assistance and optimized touch sensitivity), and feedback mechanisms (such as graphic-guided prompts), they can effectively reduce the operational complexity for older adult users (32). This reduces the frustration caused by mismatches between system design and individual capabilities, thereby alleviating the cognitive load associated with technology use.

Furthermore, adjusting the complexity of provided content according to users' knowledge understanding enables older adult users to more easily perceive the alignment between the service and their own health needs. Such “specificity” can enhance their subjective recognition of the usefulness of smart health services (16) and significantly strengthen user loyalty and dependence (62). Based on this, we propose the following hypotheses:

H5: There is a positive correlation between dynamic adaptation and (a) perceived ease of use and (b) perceived usefulness.

### 2.6 Self actualization

Self-actualization (SA) is defined as a state where an individual's physical and mental potentials are fully developed, a process often accompanied by feelings of happiness, excitement, and the fulfillment of ideals (63). When older adult users perceive that they are actively taking control of health management and related affairs through the use of smart health services—such as independently monitoring health data via intelligent devices and understanding its significance—they feel they are striving toward the ideal of a healthy older adult later life. This sense of self-actualization fosters more positive emotional perceptions of smart health services (64). They may view these services as aligned with their goals of pursuing health and a quality life, thereby developing emotional acceptance and recognition of such services (65). For older adult users, achieving a sense of self-actualization through smart health services—for instance, learning to use a health management app to record exercise achievements and witnessing improvements in their ability to maintain health—motivates them to invest more effort in learning and adapting to these services. This positive psychological feedback reduces their perception of the service's operational difficulty (66); they become more willing to overcome potential technical obstacles, thus finding smart health services easier to use.

Furthermore, when older adult users experience self-actualization through smart health services—such as communicating with experts via telemedicine (12) to obtain personalized health advice, thereby better managing chronic conditions and effectively improving health outcomes—they tangibly perceive the practical value of these services in enhancing their quality of life and maintaining health (13). This experience-based perception strengthens their recognition of the service's usefulness: they believe these services can genuinely assist them in pursuing health, independent living, and other goals, thereby enhancing their perception of the service's usefulness. Based on this, we propose the following hypotheses:

H6: Self-actualization is positively correlated with (a) perceived ease of use, (b) perceived usefulness, and (c) perceived emotion.

## 3 Research methods

### 3.1 Variable measurements

To create a contextual framework for the survey that aligns with interactions with SHS, we simulated service scenarios to help respondents better grasp the measured variables in this study. Three scenarios were constructed to mimic the context of SHS usage, and respondents were asked to recall or familiarize themselves with potential smart health service scenarios—including remote health monitoring, appointment registration services, and remote health consultations—before completing the questionnaire. This was intended to deepen their understanding of the research context. In addition, we explain how respondents would receive these services in China and the potential for different outcomes (Appendix 1).

All measurement items in this study were derived from validated scales in classic literature, with appropriate modifications to fit the specific context of this research. All questions and task scenarios were translated into Chinese to target Chinese-speaking participants. The entire questionnaire underwent a “forward-backward” translation process (67) to ensure consistency between the two languages and homogeneous understanding of the questions. Prior to the formal survey, a pilot version of the questionnaire was distributed to several researchers familiar with SHS. Based on their feedback, minor adjustments were made to the descriptions of task scenarios and measurement tools. These variables were measured using a 5-point Likert scale, where “1” indicated “strongly disagree” and “5” indicated “strongly agree.” The above constructs and their corresponding items are presented in Table 1.

**Table 1:** Questionnaire and question items.

**Constructs**	**Items**	**Sources**
Mind Interaction (MI)	MI1 I think smart health services can fully understand what I want to say.	(45, 47)
	MI2 I can fully understand the information provided by the smart health service.	
	MI3 I always get the information I need from smart health services.	
Dynamic adaptation (DA)	DA1 I feel that smart health services are understanding me more and more.	(16, 61)
	DA2 I feel that the information provided by smart health services is helping me more and more.	
	DA3 I think smart health services are getting easier and easier to use.	
Self Actualization (SA)	SA1 I feel happy to use smart health services.	(64, 65)
	SA2 I find it very rewarding to use smart health services.	
	SA3 I like to use smart health services more than ever.	
	SA4 I feel that smart health services can help me manage my health.	
Perceptual Ease of Use (PEOU)	PEOU1 Learning to use smart health services was easy for me.	(45, 51)
	PEOU2 For me, it's easy for me to get feedback on smart health services.	
	PEOU3 I find that it takes a lot of effort to use smart health services.	
Perceived Usefulness (PU)	PU1 I find smart health services useful for my health.	(14, 45)
	PU2 I feel that smart health services are good for my health.	
	PU3 I feel that smart health services can improve my health.	
Perceptual Emotion (PE)	PE1 In my life, smart health services are my partner.	(55, 56)
	PE2 I feel a sense of security when using my smart health services.	
	PE3 Smart Health Service gives me full respect and care.	
Behavioral intent (BI)	BI1 I intend to use smart health services in the future.	(10, 45)
	BI2 I plan to use the Smart Health Service.	
	BI3 I hope to use smart health services in the future.	

### 3.2 Data collection

The survey was distributed via http://www.wjx.com (a platform with a large and diverse user base, making it popular among businesses and universities) using stratified sampling by age (60–65, 66–70, 71–75, ≥76 years), educational level (junior high school and below to master's degree), and SHS usage experience (<6 months, 6–12 months, >12 months). This ensured the sample matched the demographic distribution of China's older adult SHS users (7), reducing bias from overrepresenting specific groups (e.g., highly educated older adult). To ensure that respondents met the research criteria and to enhance the accuracy of the results, a brief introduction to the definition and characteristics of SHS was provided at the beginning of the questionnaire. Respondents were instructed to carefully read the background materials and imagine themselves in scenarios involving interaction with SHS. Furthermore, a pilot survey (n = 30) was conducted with older adult SHS users to refine question clarity (e.g., simplifying technical terms like “dynamic adaptation” to “services that get easier to use over time”) and reduce response burden. Rigorous screening procedures have been implemented to exclude those who fail to understand the relevant material, complete the questionnaire in a very short time, or fail to pass the attention check (e.g., “Please select ‘Strongly agree' to confirm that you are reading carefully.”).

The formal questionnaire consisted of two parts. The first part included items measuring all constructs in the conceptual model. The second part collected respondents' demographic characteristics, including gender, age, educational level, and experience with using smart health services. To minimize the impact of common method variance (CMV), the following measures were taken during the questionnaire design phase: (1) emphasizing that the survey was completely anonymous and solely for academic research purposes; (2) distributing the questionnaire randomly online; (3) targeting respondents who were users of domestic SHS. To ensure the reliability and representativeness of the research data, respondents were required to have used at least one SHS and to answer questions based on their own usage experiences and perceptions. After collecting the questionnaires, duplicate and unqualified samples were screened and removed. Invalid questionnaires were excluded based on the following criteria: (1) those completed in less than 1 min; (2) those where the answer to the initial question “Are you currently aware of/using smart health services?” was “No.” Participants' privacy was protected, and their consent was obtained. Ultimately, 244 valid questionnaires were collected and retained for further analysis (Table 2).

**Table 2:** Demographics of respondents.

**Indicator**	**Classification**	**Frequency**	**Percentage (%)**
Gender	Man	112	45.9
	Woman	132	54.1
Age	60–65	119	48.8
	66–70	78	31.9
	71–75	34	13.9
	76 years of age and older	13	5.3
Level of education	Junior high school and below	79	32.4
	High school and equivalent	96	39.3
	University and equivalent	58	23.8
	Master's degree or above	11	4.5
Experience in use	<6 months	67	27.5
	6–12 months	75	30.7
	>12 months	102	41.8

This study adopted the minimum sample size criterion recommended by Kock and Hadaya for PLS-SEM, which stipulates that the sample size should exceed ten times the number of constructs in the model (68). With a sample size of N = 244, which far exceeds the recommended standard (N = 70), the data provide sufficient statistical power for subsequent analyses.

### 3.3 Data analysis

We analyzed the data using Partial Least Squares Structural Equation Modeling (PLS-SEM) and fuzzy-set Qualitative Comparative Analysis (fsQCA). PLS-SEM is the preferred method for explaining regression relationships among multiple variables (69), with distinct advantages that align with the needs of this study: (1) it is suitable for simultaneously analyzing complex relationships between multiple variables; (2) compared with theory testing, it is more conducive to exploratory theoretical development; (3) it requires a smaller sample size, making it applicable for estimation and testing with small samples; (4) it can handle both formative and reflective indicators; and (5) it does not require data to follow a normal distribution. Given that this study is exploratory and involves complex relationships among multiple independent variables, PLS-SEM is more appropriate for the present research.

FsQCA is used to interpret multiple plausible configurations within a holistic view of complex causal relationships, emphasizing the exploration of combined effects (70). Therefore, fsQCA is often employed to complement the analytical results of SEM in studies. The effective integration of fsQCA and SEM helps researchers overcome the limitations of linear causality and provides novel and unique insights when analyzing complex issues. Previous studies have also shown that the successful integration of these two methods can enhance the descriptive, predictive, and explanatory capabilities of scientific theories (71). Thus, while using PLS-SEM to verify the model hypotheses, this study further investigates the impact of the combined effects of various factors on users' willingness to adopt SHS through fsQCA. As it provides both variable-centric (PLS-SEM) and case-centric (fsQCA) insights (22).

## 4 Results

### 4.1 Measurement model

Preliminary data analysis revealed a KMO value of 0.944 (>0.8) and a significant Bartlett's test result (*p* < 0.001), indicating that the data are suitable for further analysis. The first stage involved evaluating the measurement model. All constructs were measured reflectively, and the measurement model of first-order constructs was assessed by examining their reliability, convergent validity, and discriminant validity. We checked the mean, standard deviation, Cronbach's alpha, composite reliability (CR), and average variance extracted (AVE) for all constructs to evaluate their reliability (Table 3). Traditionally, Cronbach's alpha and CR values are used to assess reliability. As introduced by Fornell and Larcker, both CR and Cronbach's alpha should exceed 0.60 (72). The results showed that the Cronbach's alpha values of the 7 constructs in the conceptual model ranged from 0.833 to 0.890, and the CR values ranged from 0.853 to 0.889, all exceeding the critical value of 0.60, indicating high reliability.

**Table 3:** Discriminative validity: Pearson correlation and AVE square root value.

**Elements**	**PEOU**	**PU**	**PE**	**BI**	**MI**	**DA**	**SA**
PEOU	**0.851**						
PU	0.649^***^	**0.832**					
PE	0.716^***^	0.607^***^	**0.818**				
BI	0.722^***^	0.698^***^	0.667^***^	**0.812**			
MI	0.629^***^	0.653^***^	0.586^***^	0.599^***^	**0.853**		
DA	0.420^***^	0.341^***^	0.356^***^	0.480^***^	0.358^***^	**0.814**	
SA	0.703^***^	0.634^***^	0.599^***^	0.697^***^	0.627^***^	0.412^***^	**0.794**

^***^Means significant correlation at *p* < 0.001; Bold-faced diagonal elements are the square roots of AVEs; PEOU, Perceptual Ease of Use; PU, Perceived Usefulness; PE, Perceptual Emotion; BI, Behavioral intent; MI, Mind Interaction; DA, Dynamic adaptation; SA, Self Actualization.

Subsequently, convergent validity and discriminant validity were evaluated by checking whether the item loadings on their respective constructs were sufficiently high and higher than those on other constructs (i.e., cross-loadings). Convergent validity reflects the correlation among items within the same construct. After removing one item with low loading (SA4), the remaining loadings were all higher than or close to 0.7, indicating strong convergent validity of the model. In addition, the item loadings on their respective constructs were higher than the cross-loadings (see Table 4), and the square root of AVE for all constructs was greater than their correlations with other constructs, indicating good discriminant validity (see Table 5). All correlations between variables were statistically significant. Furthermore, the variance inflation factor (VIF) for all latent constructs was calculated to test for multicollinearity. The VIF values of all items ranged from 1.447 to 4.355, all below 5, indicating no multicollinearity issue in the research data (Table 6). Finally, the Heterotrait-Monotrait (HTMT) ratio was also evaluated to test discriminant validity. The HTMT values of the sample in this study were all far below the 0.85 threshold (73). Therefore, the measurement model of this study was proven to be reliable and valid.

**Table 4:** Analysis of the necessary conditions.

**Indicators**	**BI1**		~**BI1**	
	**Consistency**	**Coverage**	**Consistency**	**Coverage**
PEOU1	0.843	0.870	0.551	0.508
~PEOU1	0.523	0.566	0.859	0.831
PU1	0.867	0.844	0.605	0.526
~PU1	0.513	0.592	0.821	0.847
PE1	0.798	0.875	0.548	0.537
~PE1	0.577	0.588	0.872	0.794
MI1	0.825	0.815	0.616	0.544
~MI1	0.539	0.611	0.791	0.802
DA1	0.798	0.778	0.640	0.557
~DA1	0.546	0.629	0.745	0.767
SA1	0.868	0.843	0.626	0.543
~SA1	0.530	0.613	0.819	0.848

**Table 5:** Cross-loadings.

**Elements**	**PEOU**	**PU**	**PE**	**MI**	**DA**	**SA**	**BI**
PEOU	0.701	0.279	0.347	0.171	0.086	0.216	0.204
	0.648	0.170	0.284	0.283	0.251	0.296	0.223
	0.732	0.222	0.263	0.238	0.179	0.256	0.168
PU	0.231	0.746	0.127	0.258	0.118	0.229	0.198
	0.136	0.724	0.270	0.207	0.129	0.264	0.197
	0.174	0.782	0.224	0.264	0.092	0.159	0.144
PE	0.208	0.211	0.746	0.248	0.131	0.186	0.167
	0.301	0.188	0.709	0.119	0.081	0.207	0.169
	0.170	0.191	0.789	0.221	0.158	0.179	0.197
MI	0.146	0.243	0.189	0.803	0.164	0.176	0.096
	0.160	0.306	0.231	0.734	0.116	0.244	0.154
	0.209	0.172	0.164	0.804	0.113	0.218	0.164
DA	0.044	0.139	0.166	0.092	0.818	0.149	0.105
	0.082	0.085	0.083	0.152	0.856	0.093	0.094
	0.168	0.042	0.040	0.060	0.862	0.062	0.126
SA	0.232	0.227	0.187	0.261	0.132	0.777	0.187
	0.209	0.191	0.249	0.187	0.146	0.805	0.091
	0.151	0.209	0.145	0.198	0.114	0.828	0.200
BI	0.200	0.273	0.246	0.242	0.210	0.163	0.734
	0.324	0.395	0.180	0.186	0.263	0.351	0.450
	0.208	0.207	0.274	0.137	0.190	0.258	0.749

Rotation method: Varimax.

**Table 6:** Scales for reliability and validity of measurement model.

**Element**	**Measurement item**	**M**	**SD**	**AVE**	**CR**	**α**	**VIF**
PEOU	PEOU1	3.361	1.137	0.723	0.887	0.886	2.935
	PEOU2	3.385	1.158				3.336
	PEOU3	3.324	1.072				2.989
PU	PU1	3.516	1.146	0.692	0.871	0.870	2.651
	PU2	3.475	1.020				2.864
	PU3	3.471	1.086				2.753
PE	PE1	3.344	1.102	0.669	0.858	0.855	2.620
	PE2	3.262	0.984				2.243
	PE3	3.254	1.070				2.833
MI	MI1	3.443	1.108	0.659	0.853	0.890	2.839
	MI2	3.475	1.135				3.254
	MI3	3.475	1.102				2.933
DA	DA1	3.385	1.267	0.728	0.889	0.853	2.220
	DA2	3.475	1.225				2.407
	DA3	3.545	1.112				2.338
SA	SA1	3.541	1.071	0.663	0.855	0.833	4.355
	SA2	3.541	1.055				3.439
	SA3	3.533	1.149				3.562
	SA4	3.299	1.167				1.447
BI	BI1	3.459	1.007	0.631	0.865	0.855	2.696
	BI2	3.463	0.957				3.097
	BI3	3.402	0.970				2.507

M, mean; SD, standard deviation; α, Cronbach's alpha; CR, composite reliability; AVE, average variance extracted; VIF, Variance inflation factor.

### 4.2 Hypothesis and model validation

**Figure 3:**
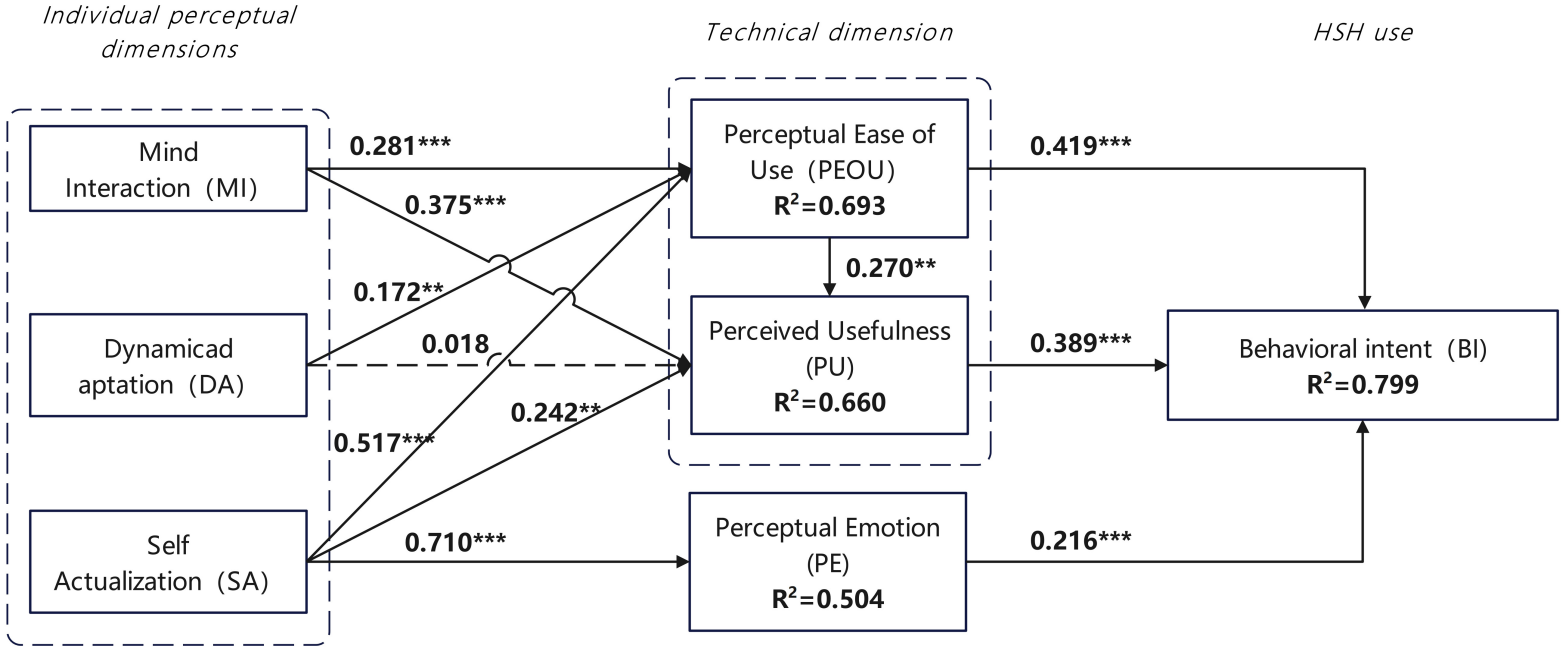
Results of structural modeling analysis.

The second stage involved evaluating the structural model. Figure 3 illustrates the PLS results of the structural modeling analysis. The *R*^2^ value assesses the explanatory power of independent variables on dependent variables. Hair et al. suggested that *R*^2^ should exceed the acceptable minimum level of 0.10, and a good explanatory power requires an *R*^2^ value greater than 0.20 (69). The *R*^2^ values of the three endogenous variables in the conceptual model were all within the acceptable range, far exceeding the minimum requirement, indicating strong explanatory power.

Specifically, among the direct effects on users' behavioral intention, perceived ease of use, perceived usefulness, and perceived emotion all exerted significant positive impacts on older adult users' behavioral intention to adopt SHS (β = 0.419, *p* < 0.001; β = 0.389, *p* < 0.001; β = 0.216, *p* < 0.001). In addition, perceived ease of use also had a significant positive effect on perceived usefulness (β = 0.270, *p* < 0.05). Therefore, H1a, H1b, H2, and H3 were verified. Mental interaction showed significant positive effects on perceived ease of use and perceived usefulness (β = 0.281, *p* < 0.001; β = 0.375, *p* < 0.001), thus verifying H4a and H4b. Dynamic adaptation had a positive effect on perceived ease of use (β = 0.172, *p* < 0.05), but its effect on perceived usefulness was insignificant (β = 0.018, *p* > 0.05). Therefore, H5a was verified, while H5b was not supported. Self-actualization exerted significant positive impacts on perceived ease of use, perceived usefulness, and perceived emotion (β = 0.517, *p* < 0.001; β = 0.242, *p* < 0.05; β = 0.710, *p* < 0.001), with the strongest effect on perceived emotion. Thus, H6a, H6b, and H6c were verified. In summary, all hypotheses in the research model were proven valid except for H5b.

### 4.3 FsQCA measurement

FsQCA is used to reveal all potential combinations of causal conditions that lead to specific outcomes. Drawing on the approach of scholars such as Eliason and Stryker (70), this study employed direct calibration to convert variables into fuzzy sets. Based on the collected data of 6 conditional variables and 1 outcome variable, the anchors for full membership, crossover point, and full non-membership were set at the 95th, 50th, and 5th percentiles, respectively. After calibration, a single-factor necessary condition analysis was conducted (71). The results in Table 4 indicate that the necessity consistency of all conditional variables is less than 0.9, which is insufficient to constitute a single necessary condition affecting users' behavioral intention (BI). This suggests that the independent explanatory power of individual conditional variables on the outcome variable is weak, and no single factor constitutes a necessary condition for BI. Therefore, it is necessary to further analyze the combined paths of each conditional variable to explore their impact on the outcome variable.

Subsequently, the threshold-calibrated data were input into the fsQCA 4.1 software, and a truth table was constructed using the “Truth Table Algorithm.” According to the analytical principles of fsQCA, the fuzzy sets composed of 6 conditional variables correspond to 2^6^ = 64 configurational paths in the truth table. In this study, the consistency criterion was set to 0.8, and the case frequency was set to 3. Data with RAW consistency greater than 0.8 and Proportional Reduction in Inconsistency (PRI) greater than 0.5 were screened, and the results showed no contradictory configurations. Finally, following the method of Ragin and Fiss (74), standardized analysis was performed using the “Standard Analyze” function in fsQCA 4.1, resulting in three final solutions. The results showed that the overall consistency (Solution Consistency) of the conditional configuration table reached 0.925 > 0.8, indicating a high degree of consistency among the selected cases; the overall coverage (Solution Coverage), including Raw Coverage and Unique Coverage, was 0.814, suggesting that the 8 configurations covered and explained 81.4% of the empirical relevance, demonstrating strong explanatory power. Overall, the conditional configuration analysis is valid. Considering that the analytical results of the fsQCA method are somewhat sensitive to the assignment method of the dependent variable (71), a further robustness test was conducted on the results. With other processing methods unchanged, the consistency threshold in the truth table was increased from 0.8 to 0.85, and the truth table was reconstructed for standardized analysis. The results remained unchanged, indicating good robustness of the findings (Table 4).

### 4.4 FsQCA results

In this study, driving users' behavioral intention toward SHS constitutes the outcome, and the causal conditions are the combinations of high and low levels of antecedent variables of behavioral intention. The fsQCA results indicate that there are 7 combinational paths that drive older adult users' behavioral intention toward SHS, and the existence of multiple sufficient configurations for behavioral intention demonstrates equifinality. Table 7 presents the coverage and consistency of the 7 configurations, where solid black circles indicate the presence of a variable, hollow circles indicate the absence of a variable, and blank cells indicate that a specific variable is not considered in the solution.

**Table 7:** Main configurations of promoting behavioral intentions.

**Configuration**	**Solution**						
	**Q1**	**Q2**	**Q3**	**Q4**	**Q5**	**Q6**	**Q7**
PEOU	⦁	⦁	⦁	⦁	⊗	⊗	⦁
PU	•		•	⊗	⦁	⦁	⦁
PE	•	•		⊗	⊗		•
MI		•		⊗	⦁	⦁	⦁
DA			•		⦁	⊗	⦁
SA	•	•	•	•		⦁	
Consistency	0.962	0.957	0.968	0.971	0.965	0.956	0.970
Raw coverage	0.658	0.634	0.624	0.314	0.359	0.334	0.567
Unique coverage	0.009	0.017	0.024	0.019	0.024	0.014	0.014
Solution coverage	0.814						
Solution consistency	0.925						

The black circles $\frac{1}{4}$ presence of the variable; hollow circles $\frac{1}{4}$ absence of the variable; blank $\frac{1}{4}$ not considered in the solution.

The fsQCA analysis confirms the results of the PLS-SEM analysis, proving that combinations of multiple factors are predictors of behavioral intention. Solution 1 (Q1) exhibits high consistency (0.962) and explains many cases (coverage = 0.658), highlighting the key factor combination for behavioral intention. This means that perceived ease of use, perceived usefulness, perceived emotion, and self-actualization are critical factors driving users' behavioral intention to adopt SHS. The remaining solutions show a high level of consistency. In addition, Q2, Q3, and Q4 further confirm the important roles of perceived ease of use and self-actualization in Q1. Q5 suggests that when perceived ease of use and perceived emotion are absent, the combination of high levels of perceived usefulness, mental interaction, and dynamic adaptation can also promote behavioral intention toward SHS, which may reflect the complementary relationship between the two systems. Both Q5 and Q6 have relatively low coverage (0.359, 0.334), indicating that the absence of perceived ease of use and perceived emotion has a significant impact on promoting users' behavioral intention to use SHS, even though users can benefit from other aspects of SHS. Q7 shows that in the absence of self-actualization, more comprehensive elements are required to promote users' behavioral intention toward SHS.

## 5 Findings

This study systematically reveals the behavioral driving mechanisms behind older adult users' adoption of Smart Health Services (SHS) by integrating the Technology Acceptance Model (TAM) and Emotional Durable Design (EDD) theory, combined with PLS-SEM and fsQCA methods. The results of data analysis address the research questions. Firstly, the synergistic effect of technological and emotional dimensions forms the foundation for older adult users' adoption of SHS. PLS-SEM results show that perceived ease of use (β = 0.419, *p* < 0.001), perceived usefulness (β = 0.389, *p* < 0.001), and perceived emotion (β = 0.216, *p* < 0.001) all directly and positively influence behavioral intention, verifying the applicability of TAM's core variables in the older adult population (24, 50). Meanwhile, mental interaction (on perceived ease of use: β = 0.281, *p* < 0.001; on perceived usefulness: β = 0.375, *p* < 0.001) and self-actualization (on perceived emotion: β = 0.710, *p* < 0.001) in emotional durable design exert indirect effects on behavioral intention through internalization processes, indicating that emotional factors are not independent of technological factors but function by reshaping users' cognition of technological value (41, 43).

Secondly, fsQCA analysis further reveals the complexity of variable configurations. The seven valid paths indicate that high behavioral intention can be achieved through diverse combinations of factors (equifinality): when perceived ease of use and self-actualization coexist (Q1–Q4), high behavioral intention can still be formed even if some variables (e.g., dynamic adaptation) are absent; in contexts where perceived ease of use is lacking (Q5, Q6), high perceived usefulness needs to be combined with mental interaction or dynamic adaptation to compensate for deficiencies in technological experience. This finding supplements the net effect analysis of PLS-SEM, suggesting that the impact of a single variable should be understood within a framework of multi-factor interactions (22, 74). Additionally, self-actualization, as a core variable in the emotional dimension, shows strong influence in both analyses: in PLS-SEM, its path coefficient on perceived emotion (β = 0.710) is the largest among all relationships; in fsQCA, 6 out of 7 configurations include high self-actualization. This indicates that older adult users' adoption of SHS is not merely an instrumental choice but also a fulfillment of emotional needs for health autonomy and self-worth realization (63, 64). By integrating emotional durable theory and the TAM framework, these factors can drive users' behavioral intention toward SHS through both emotional and technological dimensions of SHS.

### 5.1 Theoretical implications

Our research provides several theoretical contributions. Firstly, this research expands the emotional explanatory dimension of technology acceptance theory. Existing studies have predominantly focused on the technological attributes of the TAM model (e.g., perceived ease of use, usefulness) (23, 28), while this study, by integrating EDD theory with TAM, reveals that emotional factors (e.g., mental interaction, self-actualization) not only independently affect behavioral intention but also form a “technology-emotion” synergistic effect by regulating technological perceptions (e.g., self-actualization influences perceived ease of use, β = 0.517). This finding offers an emotional durability perspective into the technology acceptance model, explaining why some technologically advanced but emotionally underdesigned SHS are difficult to be accepted by older adult users (10, 20).

Secondly, this study develops a dual-perspective analytical framework of “variable net effect-configurational effect.” PLS-SEM analysis reveals the direct and indirect effects of individual variables (e.g., perceived ease of use influences perceived usefulness, β = 0.270), while fsQCA identifies differentiated paths such as “high self-actualization + high perceived emotion” (Q1) and “high mental interaction + high perceived ease of use” (Q2), confirming the “equifinality” characteristic of complex behavioral intentions (22, 74). This methodological integration divorces from the oversimplification of causal relationships in single quantitative methods, provideing a more comprehensive theoretical toolkit for understanding the complexity of older adult users' technology adoption.

Furthermore, this study refines the emotional mechanism of technology adoption among the older adult. Unlike young groups who prioritize technological functions (14), the analsyis reveals that the emotional needs of older adult users are distinctive: self-actualization enhances emotional identification through strengthening “health control” (β = 0.710), while mental interaction reduces technological alienation through “narrative transmission” and “two-way dialogue” (β = 0.375). This finding supplements the understatement of emotional heterogeneity in older adult technology acceptance research (11, 12), providing a complementary perspective for developing older adult-friendly technology theories.

### 5.2 Practical implications

**Figure 4:**
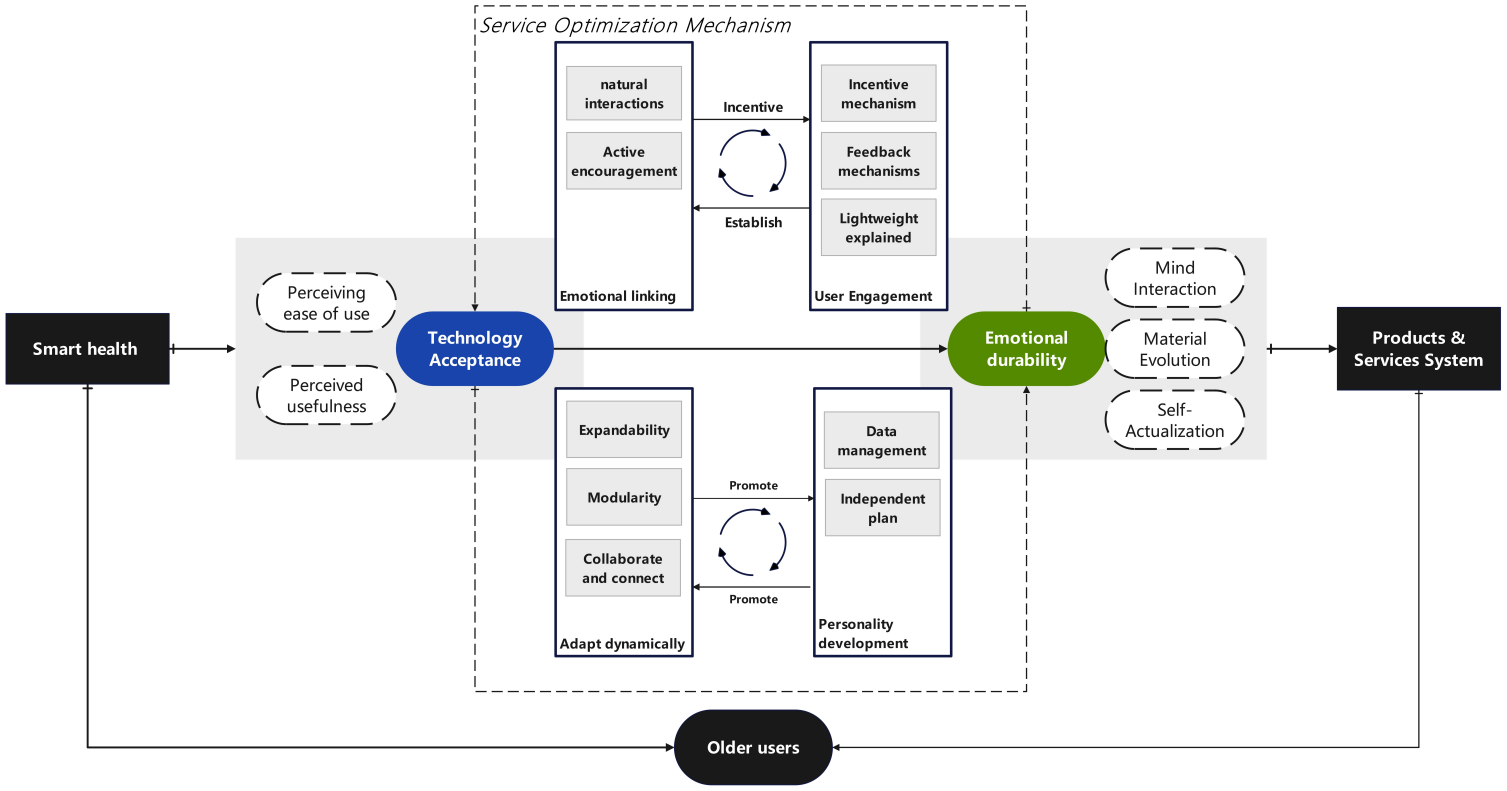
Mechanism to promote user behavioral intent using SHS.

The findings of this study further provide specific practical guidance for the design, optimization, and promotion of smart health services. Figure 4 outlined the mechanism to promote user behavioral intent using SHS. The outer layer of the model shows the interaction process and influencing factors between smart health services and older adult users, while the inner layer presents key design strategy mechanisms. Firstly, at the technical design level, it is necessary to establish a dual-oriented standard of “ease of use-emotionalization.” Based on the results of PLS-SEM analysis, perceived ease of use (β = 0.419) is a core variable directly affecting behavioral intention. It is recommended to reduce the usage threshold by simplifying operation processes (e.g., voice interaction instead of touchscreen operation) and providing visualized health data (e.g., dynamic charts instead of text reports) (7, 16). Meanwhile, combined with the strong influence of self-actualization (all Q1–Q4 include high self-actualization), a “health achievement system” can be designed (e.g., recording medication adherence points, rehabilitation training progress) to strengthen the willingness to use continuously through a sense of achievement (64). Aligning with Zhou (64) use of self-actualization to drive exercise system adoption. In addition, natural language processing technology can be adopted to enable users to conduct natural conversations with products through voice, rather than just simple command input. For example, Karim et al. (75) strengthened users' willingness to use through prototype testing of “anthropomorphism” and emotional expression of care companion robots.

Secondly, at the service configuration level, it is necessary to provide differentiated solutions for different user characteristics. The paths identified by fsQCA indicate that for older adult people with low technological familiarity (low perceived ease of use), acceptance can be improved through the combination of “high mental interaction + high dynamic adaptation” (Q5). For instance, exclusive health consultants can be assigned for real-time interactive guidance, or service content can be dynamically adjusted based on user feedback (e.g., dialect adaptation, slower rhythm) (43, 49); for users with high emotional needs, the synergy of “perceived emotion + self-actualization” (Q1) should be strengthened, such as enhancing emotional connection through anniversary health reminders and family-shared health reports (42). Furthermore, functional configurability can be achieved through a modular architecture, such as presetting a “minimalist mode” for the oldest-old and opening advanced options like “custom health goals” for the young-old. Chatrati et al. (76) dynamically optimized the home health monitoring system, which automatically adjusts monitoring frequency and generates customized health plans by analyzing users' living habits and physiological data. For hypertensive patients, the system links with community hospitals to send medication reminders after detecting abnormal data and recommends low-salt recipes through algorithm optimization. Meanwhile, the system supports functional expansion, such as connecting to smart home devices to achieve adaptive environmental regulation (temperature, humidity, lighting), meeting users' diverse needs. Through technological evolution and ecological collaboration, services are transformed from “generalization” to “precision.”

Last but not least, at the promotion strategy level, it can be beneficial to develop a communication system of “technological empowerment-emotional resonance.” To address older adult users' distrust of technology (9), community pilots can be used to demonstrate the practical value of SHS in reducing family care pressure (perceived usefulness) and improving independent living ability (self-actualization); meanwhile, users can be invited to participate in service optimization (e.g., co-designing health monitoring indicators) to enhance identification through “creative participation” in mental interaction (43, 45). In addition, a two-way feedback channel can be established to allow users to confirm or fine-tune system adjustments, thereby enhancing their sense of control over product functions and emotional resonance, and promoting the transformation of smart health care systems from “instrumental rationality” to “emotional partners.”

### 5.3 Limitations and future research

This study contends with several limitations. Firstly, the sample has limited representativeness. The research sample primarily consists of older adult users who have experience with SHS (244 valid questionnaires), with the 60–70 age group accounting for 80.7% (Table 2). This may not represent older adults aged 75 and above or those with zero exposure to technology. Future studies could adopt stratified sampling to expand the sample scope, including older adult individuals in rural areas and those with low educational levels, to test the cross-group applicability of the model. Secondly, the cross-sectional research design is limited in capturing long-term usage dynamics. Older adult users' acceptance of SHS may change over time with factors such as health status (e.g., worsening chronic conditions may increase dependence). Longitudinal studies are recommended to track changes in user perceptions (e.g., perceived usefulness) and behavioral intentions over 6–12 months or longer timeframe, revealing the temporal nature of causal relationships. Finally, the generalizability of configurational analysis requires further validation. The seven paths identified by fsQCA are based on a specific cultural context (China's home-based older adult care scenario), while older adult care models in other countries (e.g., Western community-based care) may influence variable configurations (2). Cross-national comparative studies could be conducted to explore how cultural factors (e.g., collectivism vs. individualism) moderate configurational paths, enhancing the global applicability of the theory. Future research can further explore two areas: first, exploring the role of intergenerational support in SHS adoption, such as whether assistance from children moderates the impact of emotional factors; second, introducing technology anxiety as a boundary condition to analyze how it interferes with the “technology-emotion” interaction effect.

## 6 Conclusion

Drawing on EDD theory, this paper provides more comprehensive understanding of the behavioral intentions of SHS users. The behavioral intentions of older adult users toward SHS are the result of the synergistic effect of technical attributes and emotional factors. In the technical dimension, perceived ease of use and perceived usefulness are direct driving factors; in the emotional dimension, self-actualization plays a key role by strengthening emotional identification, and mental interaction exerts a key function by optimizing technical perception. fsQCA further confirms that high behavioral intentions can be achieved through diverse combinations of factors, such as the paths of “perceived ease of use + self-actualization” and “perceived usefulness + mental interaction,” highlighting the equifinality characteristic in complex causal relationships. Finally, we propose specific strategies for the humanized development of SHS. The two-dimensional framework advances human-centered design in geriatric technology by explicitly integrating emotional persistence beyond the “function-first” paradigm. This paper deepens the understanding of older adult users' technology adoption behavior, strengthens the theoretical understanding of SHS behavioral intentions, expands the application scope of relevant theories, and provides valuable guidance for SHS practitioners.

## Data Availability

The original contributions presented in the study are included in the article/Supplementary material, further inquiries can be directed to the corresponding author.
