# Supplementary material for: Exploring behavioral factors and emotional mechanisms underlying older adult users' adoption of smart health services: evidence from PLS-SEM and fsQCA
Source: Front Public Health. 2025 Sep 26;13:1673340. doi: 10.3389/fpubh.2025.1673340 (PMC12512665; doi:10.3389/fpubh.2025.1673340)
Supplement: Supplementary file 1 [file Table_1.docx]

**Annex 1：**

“*In China, the delivery of SHS for home-based elderly care is characterized by a state-led, community-embedded system, which aligns with the national strategy of ‘active aging’ (Han et al., 2020; Zhang et al., 2020). Most SHS accessed by the study’s respondents are integrated into public elderly care networks: for example, community health centers provide free or subsidized remote health monitoring devices (e.g., blood pressure monitors linked to local hospitals), and public hospitals offer state-funded teleconsultation services via government-developed platforms (e.g., the ‘National Healthcare Big Data Platform’). This state-provided model ensures broad accessibility—especially for low-income or rural elderly—and builds high initial trust, as users perceive services as non-commercial and aligned with public health goals (Chen et al., 2022).*

*In contrast, market-provided SHS (e.g., commercial health management apps, private telemedicine services) remain less prevalent among the study’s respondents (60+ years old), primarily due to concerns about cost, data privacy, and technical complexity (Liu & Tao, 2022). If the sample had included users of market-provided SHS, attitudes toward perceived usefulness and perceived emotion might differ: market services often emphasize personalization (e.g., tailored health advice for high-income groups) but may trigger distrust due to profit motives (Kang et al., 2022). However, the core mechanism identified in this study—the synergy between technological attributes (perceived ease of use/usefulness) and emotional factors (self-actualization, mental interaction)—remains generalizable. State-provided services strengthen this synergy through trust, while market services would need to address emotional barriers (e.g., privacy concerns) to achieve similar acceptance. This contextual difference highlights the need for context-specific SHS design, which is a key contribution of our study to global elderly technology acceptance research.*”

**References**

Chen, Y., Zhang, L., & Wei, M. (2022). How does smart healthcare service affect resident health in the digital age? Empirical evidence from 105 cities of China. Frontiers in public health, 9, 833687.

Han, Y., He, Y., Lyu, J., Yu, C., Bian, M., & Lee, L. (2020). Aging in China: Perspectives on public health. Global[J].Health Journal, 4(1),11–17.

Kang, H. J., Han, J., & Kwon, G. H. (2022). The acceptance behavior of smart home health care services in South Korea: an integrated model of UTAUT and TTF. International Journal of Environmental Research and Public Health, 19(20), 13279.

Liu, K., & Tao, D. (2022). The roles of trust, personalization, loss of privacy, and anthropomorphism in public acceptance of smart healthcare services. Computers in Human Behavior, 127, 107026.

Zhang, Q., Li, M., & Wu, Y. (2020). Smart home for elderly care: development and challenges in China. BMC geriatrics, 20(1), 318.
